# Reversible optical data storage and encryption enabled by phase-change and hydrogel integration

**DOI:** 10.1038/s41377-026-02330-5

**Published:** 2026-05-18

**Authors:** Asad Nauman, Guli Gulinihali, Tristen Moncada, Muhammad Waleed Khalid, Tristan Tjussardi, Yeshaiahu Fainman, Abdoulaye Ndao

**Affiliations:** Department of Electrical and Computer Engineering, University of California, San Diego, La Jolla, CA USA

**Keywords:** Nanocavities, Polymers

## Abstract

Phase-change materials and hydrogels, which are emerging as versatile, low-cost, high-speed materials with large-area processing capabilities, are key building blocks for next-generation optical information storage and multi-level encryption. Here, we introduce a hybrid platform that synergistically integrates directly laser-written antimony trisulfide (Sb₂S₃) with a humidity-responsive azido-grafted carboxymethyl cellulose (CMC-N₃) hydrogel, enabling the fabrication of a full-color image multiplexing. The Sb₂S₃ medium layer enables non-volatile, rewritable optical data via laser-induced amorphous–crystalline transitions, while the hydrogel introduces UV-programmable cavity modulation for data writing and consequently, achieving a humidity-dependent tunable full-color image response. Together, these dynamic and reversible processes enable independent encoding and retrieval of multi-level information, resulting in a transmissive multiplexed optical storage device. This multi-programmable layer approach establishes a new paradigm for multifunctional optical devices, unlocking opportunities in secure data storage, anti-counterfeiting displays, and environmental sensing.

## Abstract (web summary)

A Programmable Hybrid Platform for On-Demand Data Storage and Dynamic Optical Displays: Crucial for date storage and encryption, this platform advances data storage and dynamic display by integrating light-written phase-change materials with hydrogels. Modulating humidity dynamically alters the displayed visuals, seamlessly revealing or concealing the stored information.

## Graphical abstract

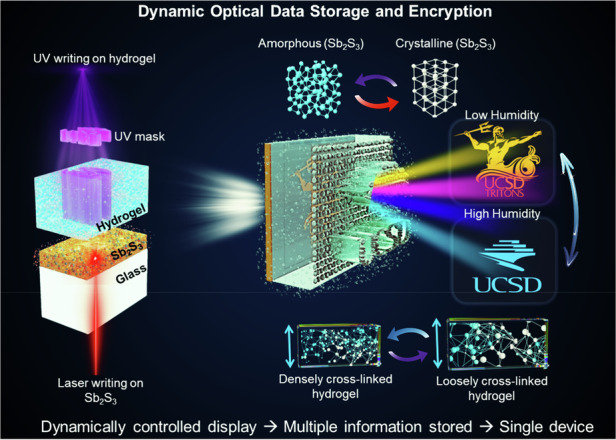

## Introduction

The exponential growth of the information age has created an urgent demand for storage technologies that combine ultra-high capacity with secure and durable media. Optical storage, with its inherent advantages of high density and cost-effectiveness, represents a compelling alternative to conventional electronic approaches.^1–5^ However, traditional optical storage systems remain fundamentally limited by the diffraction limit of light.^6,7^ Overcoming this constraint is critical for advancing nanoscale data storage. The advent of far-field super-resolution microscopy, which circumvents the diffraction limit through controlled emission modulation, has reinvigorated interest in optical strategies for high-density information storage.^8^ Building on these advances, multiplexed optical storage media have emerged as a powerful route to dramatically expand storage capacity and ensure long-term data stability.^9–11^ Despite this promise, existing multilayer data storage platforms face persistent limitations, including restricted reconfigurability, lack of dynamic full-color modulation, and often reliance on photochromic materials with finite lifecycles.^12–14^

Advances in nanophotonics have enabled remarkable control over light–matter interactions,^15–18^ giving rise to vivid structural coloration rather than the use of pigments or dyes.^19–22^ Over the years, advanced photonic designs leveraging dielectric and plasmonic effects have enabled striking structural colorations using multilayer thin films,^23,24^ high-index resonant disks,^25^ vertical nanopillars,^26,27^ and subwavelength metallic grooves.^28,29^ Expanding upon these concepts, metasurface-based optical data storage has emerged as a powerful platform, where multiplexed holographic information can be recorded and reconstructed with unprecedented density and redundancy.^30–32^ Such functionality is typically realized through interleaved or multilayer metasurface architectures, in which subwavelength elements within each pixel independently modulate phase, enabling wavelength-selective switching.^33,34^ Extensions exploiting polarization,^35,36^ orbital angular momentum,^37–39^ and angle-dependent^40^ multiplexing have further expanded information capacity, yielding multiple independent image channels from a single metasurface plane.

Yet these existing image multiplexing techniques based on metasurfaces are fundamentally limited by their dependence on narrowly defined illumination conditions. Specifically, these implementations typically rely on narrowband, polarization-controlled, or angle-specific excitation, which restricts both practical accessibility and large-scale applicability. Furthermore, current implementations of image multiplexing often rely on anisotropic plasmonic metasurfaces^41^, which are fabricated using high-resolution techniques such as electron beam lithography or focused ion beam. While these fabrication approaches offer nanometer-scale precision, they are inherently costly and low-throughput, rendering them unsuitable for scalable manufacturing or customizable deployment. Recently, hydrogels and phase-change materials have been explored as promising media for full-color optical data storage, wherein light serves as the data writing medium. Hydrogels undergo volumetric and refractive index changes when interacting with moisture,^42^ thereby altering its optical response, while, Sb₂S₃, typical alloys in the chalcogenide family, are suitable for visible photonics owing to its high index contrast of ~1, good stability under room temperature, wide bandgap, and relatively low loss compared to generally employed phase-change materials (PCMs) in the visible.^43–45^

Here, we report an image multiplexing strategy that overcomes the stringent illumination requirements and fabrication constraints of conventional metasurface-based approaches. Our design leverages structural color cavities that integrate independently reconfigurable antimony trisulfide (Sb₂S₃) and azido-grafted carboxymethyl cellulose (CMC-N₃) layers, enabling direct image retrieval under ambient lighting without the need for auxiliary optics or user training. Distinct information is encoded in Sb₂S₃ and CMC-N₃ layers and retrieved with full-color differentiation under defined humidity conditions, ensuring robust and intuitive readout. Re-writability is achieved through complementary control mechanisms: focused laser pulses induce reversible phase transitions in Sb₂S₃, while patterned ultraviolet irradiation encodes CMC-N₃ hydrogel layer. This scalable and low-cost strategy circumvents electron-beam lithography, delivering a compact, rewritable, multi-color optical storage platform with direct relevance to data security, anti-counterfeiting, and consumer display technologies.

## Results

**Fig. 1:**
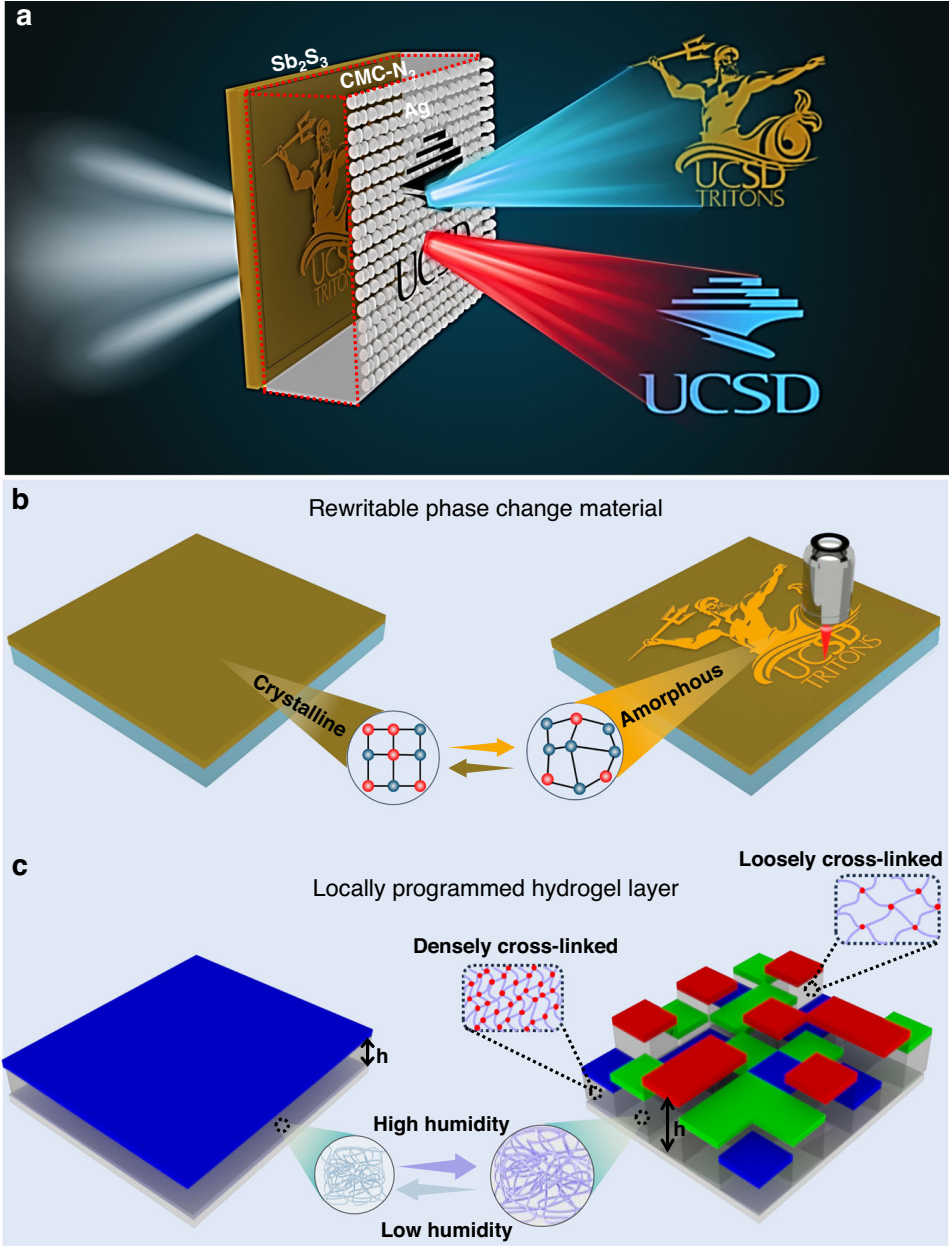
Schematic and principle of multiplexed optical information. **a** schematic illustration of our proposed reversible optical storage device displaying humidity-responsive distinct information. The device consists of a multilayer resonator cavity composed of 25 nm Ag nanoislands, a 160 nm CMC-N_3_ hydrogel, and a 20 nm Sb₂S₃ phase-change film. The information encoded on the Sb₂S₃ (UCSD Triton logo) is visible in a low-humidity state, while the information encoded on the CMC-N_3_ hydrogel (UCSD library logo) remains encrypted. The UCSD library logo is retrieved at a high humidity state, manifested by the localized swelling of CMC-N_3_, which encrypts the information on Sb₂S₃. **b** Demonstrates the data encoding on Sb₂S₃ layer where a focused nanosecond pulsed laser with short duration and high-intensity pulses is utilized to switch the thermally annealed c-Sb₂S₃ to a-Sb₂S₃ state, while thermal annealing or a continuous wave (CW) laser is used to erase the information. **c** Schematic of the hydrogel cavity under low and high humidity environments, where the chemical structure of CMC-N_3_ with different cross-linking densities exhibits distinct swelling ratios. At lower humidity levels, CMC-N_3_, with uniform thickness throughout the device, filters a uniform blue color under incoherent white light illumination. At higher humidity, the swelling ratios, depending on the cross-linking density of the CMC-N_3_, result in varying cavity thicknesses, allowing for modulated color filter ability positionally

To achieve full color optical information multiplexing, a resonator cavity with independently programmable thin layers capable of encoding distinct optical information is implemented. The fabricated structure comprises Sb₂S₃ thin film, CMC-N_3_ hydrogel, and silver (Ag) nanoislands. The Sb₂S₃, an optically reconfigurable phase change material,^46^ is deposited as a thin film on a transparent glass substrate, allowing for the direct laser writing of information from both the top and bottom sides, thereby providing flexibility in data encoding. The hierarchically encoded information can be retrieved independently in transmission mode by modulating external relative humidity (Fig. 1a). Due to the significant refractive index difference between the amorphous (a-Sb₂S₃) and crystalline (c-Sb₂S₃) states of Sb₂S₃ (Supplementary Fig. S1), high-contrast optical information is printed on the PCM layer. The data is written on the c-Sb₂S₃ layer via nanosecond optical pulses (λ = 527 nm) (Supplementary Fig. S2). A short and high-intensity pulse is utilized to switch the c-Sb₂S₃ to a-Sb₂S₃, while a continuous wave (CW) laser or heat can be used to switch the a-Sb₂S₃ to c-Sb₂S₃ (Fig. 1b). This capability allows repeated writing and erasing of information on the same Sb₂S₃ film region, resulting in an independent device that can display and erase multiple prints. Subsequently, a CMC-N_3_ hydrogel layer is spin-coated on Sb₂S₃ and geometrically patterned by selectively exposing the hydrogel to UV irradiation using a photomask to control the cross-linking density positionally (Fig. 1c). By depositing a thin layer of disordered Ag nanoislands, we demonstrate a humidity-responsive optical information encoding and anti-counterfeiting device that exhibits diverse optical responses. The surface morphology of the deposited Ag layer (Supplementary Fig. S3) confirms that the Ag layer consists of a discontinuous nanoislands structure, which remains locally deformable and does not mechanically inhibit the swelling of the underlying hydrogel. Owing to the controlled cross-linking density of CMC-N_3_ and the refractive index modulation of Sb₂S₃, a full color optical response is achieved. For instance, under low humidity conditions (RH ~ 40%), the patterned CMC-N_3_ layer with highly and loosely cross-linked regions remains in a non-swollen state, preserving a uniform cavity thickness (*h*), ensuring a plasmonic cavity with the same effective optical path across the entire sample. Consequently, the information encoded on the Sb₂S₃ layer is retrieved (Fig. 1a). Upon exposure to high humidity (RH ~ 85%), the information encrypted in the CMC-N_3_ layer is decrypted by adjusting the optical path lengths independently based on the preprogrammed patterns (Fig. 1c). The information printed on the Sb₂S₃ layer becomes unclear due to the high contrast difference between the layers as the humidity increases. This humidity-triggered optical information storage is fully reversible, allowing dynamic concealment and display of encrypted patterns based on environmental conditions. The synergy between the programmable hydrogel and the rewritability of Sb₂S₃ enables a multifunctional platform for secure optical data storage, encryption, and anti-counterfeiting.

**Fig. 2:**
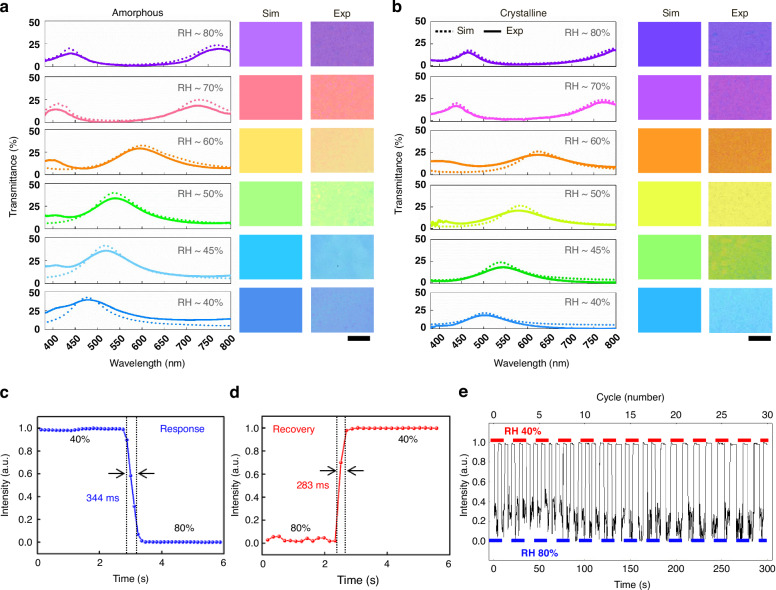
Optical behavior of the proposed plasmonic cavity. Experimental and simulated transmission spectra and corresponding structural color images of our proposed humidity-responsive device incorporating **a** amorphous and **b** crystalline Sb₂S₃ layers without encoding information. Simulated (dotted lines) and experimental (solid lines) spectra show excellent agreement with observed color outputs. The scale bar is 500 µm. **c** the dynamic humidity response of the CMC-N₃ hydrogel-based resonator, as measured by the transmitted intensity, shows a fast response (344 ms) to humidity increases from RH ~ 40% to RH ~ 80%. **d** The recovery (283 ms) between relative humidity RH ~ 40% to RH ~ 80%. **e** The repeatability over 30 switching cycles modulated between RH ~ 40% to RH ~ 80%

To demonstrate the synergistic optical behavior of hydrogel swelling and the phase change between the amorphous and crystalline states of Sb₂S₃, a cavity with no information (uniform cross-linking density of CMC-N_3_) encoded is fabricated. A disordered subwavelength plasmonic layer is incorporated on top of the hydrogel. During electron-beam (E-beam) evaporation of Ag film, due to self-aggregation, Ag forms randomly distributed self-assembled nanoislands that act as localized plasmonic scatterers. Surface structural characterization was carried out using scanning electron microscopy (SEM) and atomic force microscopy (AFM) to clarify the morphology of the Ag nanoislands and to provide accurate structural parameters for subsequent electromagnetic simulations (Supplementary Fig. S3). These nanostructures not only serve as a porous structure to allow the water molecules to bond to the hydrogel but also support broadband localized surface plasmon resonances, leading to enhanced electric field confinement and strong spectral coloration without requiring complex nanofabrication (Supplementary Fig. S3). While the cavity operates primarily through interference-based modulation of the optical path length, the disordered Ag layer introduces plasmonic absorption, contributing to the observed color saturation and contrast. Full-wave electromagnetic simulations confirm that the interaction between the plasmonic nanoislands and the hydrogel cavity results in enhanced optical field localization and robust spectral tuning under humidity-induced thickness changes. This hybrid structure thus combines the spectral selectivity of a Fabry–Pérot cavity with the broadband enhancement of a disordered plasmonic interface. Since plasmonic cavity resonance depends on both cavity thickness and refractive index of the substrate, this strategy enables additional tunability, manifested by hydrogel and Sb₂S₃ layer. Figure 2a, b demonstrates both simulation and experimental dynamic, humidity-sensitive optical properties of our designed plasmonic cavities, comparing the optical response in both amorphous and crystalline states of Sb₂S₃ at a fixed CMC-N_3_ thickness of 150 nm under relative humidity (RH) varying from ~40% to ~80%. The optical images and resonant spectra were measured in a controlled-humidity chamber (Supplementary Fig. S4). The refractive index of the hydrogel decreases with increasing relative humidity due to water uptake, thereby lowering its optical density (Supplementary Fig. S5). This reduction in refractive index leads to a blue-shift in the plasmonic cavity resonance. However, this effect occurs simultaneously with thickness swelling of the hydrogel (Supplementary Fig. S6), which increases the cavity thickness and causes a red shift in the resonance wavelength. These two effects act in opposite directions, but the magnitude of the thickness change dominates the overall optical path length modulation (Supplementary Fig. S7). As a result, the net spectral shift trends toward longer wavelengths with increasing RH. While the trade-off between decreasing refractive index and increasing cavity thickness slightly moderates the red-shift rate, it does not qualitatively alter the optical behavior of the device. Furthermore, the Sb₂S₃ layer, which switches between amorphous and crystalline forms, introduces a significant refractive-index contrast (Δn ~1.0) with relatively low visible spectrum absorption, as reflected by the extinction coefficient (Supplementary Fig. S1), resulting in noticeable spectral shifts at constant hydrogel thickness. Even though the losses in Sb_2_S_3_ in the visible are not negligible, in optical color display or image encoding systems, moderate losses may not necessarily be limiting, what matters more is color contrast and range. Finite-difference time-domain (FDTD) simulations yield color swatches that closely match experimental results, resulting in a broad RGB gamut due to hydrogel swelling (Supplementary Fig. S8).

Time-resolved measurements indicate rapid and reversible optical tuning, with response and recovery times of ~344 ms (Fig. 2c) and ~283 ms (Fig. 2d), respectively, between 40% and 80%. The measured responses and recovery times are defined as the time required to reach 90% equilibrium intensity (T_90_) from 10% at the initial state (T_10_) or vice versa, for CMC-N_3_ films with plasmonic cavities. This rapid response is attributed to the disordered Ag nanoislands, which provide more exposed sites for water molecules to bind to the hydrogel. The nanoscale gaps and discontinuities between the Ag nanoislands facilitate rapid vapor diffusion and localized hydration, reducing the diffusion barrier and accelerating the swelling kinetics of the hydrogel layer. In contrast, a continuous Ag film forms a dense barrier that restricts water vapor diffusion into the hydrogel, thereby slowing the humidity response and limiting the dynamic performance of the device. Durability tests over 30 humidity cycles in a controlled chamber confirm stable operational performance with negligible degradation (Fig. 2e). The cross-linking points, formed by strong covalent bonds, demonstrated robust stability under repeated swell/de-swell cycles, showing no signs of degradation across tens of experimental repetitions.

**Fig. 3:**
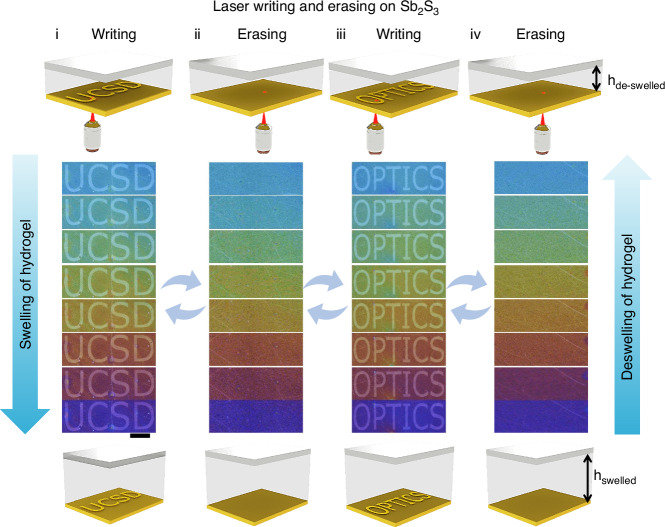
Humidity responsive dynamic full color and rewritable display. Schematic and corresponding optical microscopic images of the cavity under different humidity states for color variation of laser-printed text information on Sb_2_S_3_ layer showing (i) printed UCSD with dynamic color response from top to bottom at different humidity level, (ii) erased optical information under different humidity level (iii) re-printed the word OPTICS with dynamic color response from top to bottom at different humidity level, and (iv) erased optical information under different humidity level. The scale bar is 1 mm

Color nano-print devices based on Sb₂S₃ films face the challenge of dynamic color variation once they are fabricated.^46^ Our device design integrates hydrogel to mitigate the challenges of the static behavior of phase change materials post fabrication. Figure 3 illustrates a hydrogel-integrated optical resonator with Sb₂S₃ capable of spatially multiplexed information encoding and reversible humidity-dependent switching. Optical messages (“UCSD” and “OPTICS”) are independently encoded into the Sb₂S₃ layer via a focused nanosecond pulse laser, enabling spatially resolved modulation of the refractive index. Each region functions as a discrete plasmonic cavity with a distinct optical resonance condition, controlled by both the phase of Sb₂S₃ and the swelling-induced variation in cavity thickness of the hydrogel layer. When exposed to varying humidity levels, the hydrogel expands, red shifting the resonance condition and revealing a dynamic transition of colors from blue to red across both encoded patterns. Optical contrast and visibility of each message evolve systematically, governed by the humidity-induced cavity tuning and the inherent optical properties of the underlying Sb₂S₃.

Color switches are obtained under ambient light illumination, confirming clear and vivid color transitions, with experimental results in strong agreement across multiple humidity levels. Importantly, each pattern can be independently written, erased, or modified by light stimuli, while its visibility and coloration remain actively modulated by ambient humidity, enabling a hybrid photonic memory platform with both permanent and dynamic addressability. Here, it is noticeable that the write/erase is performed by illuminating through the glass substrate, and the beam is focused precisely onto Sb_2_S_3_ layer using a microscope objective. This geometry confines the highest optical intensity and the associated photothermal heating to the Sb_2_S_3_ film. Because the hydrogel layer is located above the Sb_2_S_3_ layer, it is out of the focal plane and therefore experiences a substantially lower fluence. In addition, erasing is performed using short, localized laser exposures, which limit thermal diffusion into the overlying hydrogel. Under these conditions, we did not observe any visible change in the hydrogel UV-patterned features after PCM write/erase operations. Such synergy between phase-change optics and hydrogel mechanics presents new opportunities for adaptive displays, anti-counterfeiting systems, and humidity-aware encryption.

**Fig. 4:**
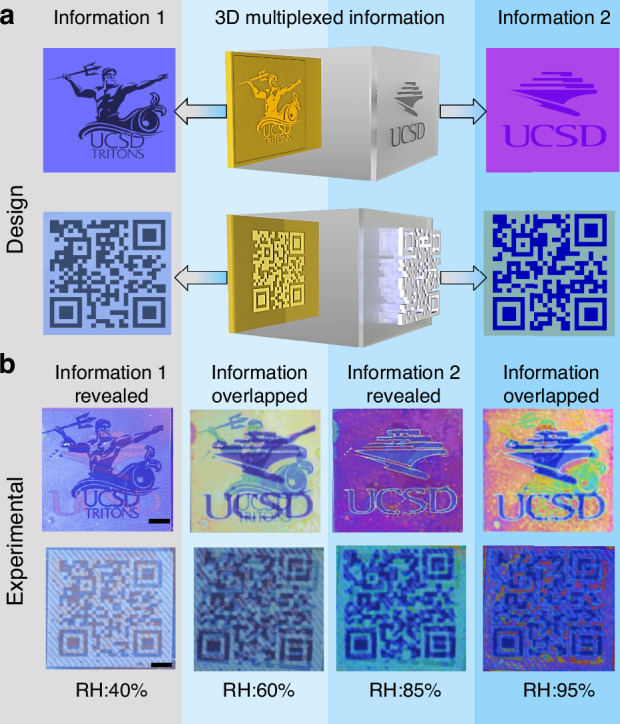
Dynamic image transformation for anti-counterfeiting displays and optical storage. **a** Schematic demonstration of a humidity-responsive, reversible dynamic data encryption and retrieval mechanism with an independently programmable display layer for stack-multiplexed information storage. **b** The transformation of the UCSD Triton logo and QR code leading to the Ndao lab website (laser printed on the Sb_2_S_3_ layer) to the UCSD library logo and QR code leading to UCSD website (patterned on the CMC-N_3_ layer). The information encoded on the Sb_2_S_3_ layer can be clearly read out at a 40% humidity level, while the information encoded on the CMC-N_3_ layer can be read out at a 85% humidity level (indicated in green dotted lines). The information in other states remained multiplexed or deteriorated due to high humidity. The scale bar is 150 µm

Utilizing the independent and simultaneous programmability of Sb₂S₃ and local geometrical modulation of CMC-N₃, the designed device enables full-color multiplexed optical information for transformative anti-counterfeiting displays. Figure 4a demonstrates the design and operation of an optical encryption system where multiple information are spatially encoded that can be decoded reversibly by humidity-induced optical modulation. The platform combines direct laser-written information on Sb₂S₃ phase-change films by modulating the transition between amorphous and crystalline states using laser. On top of a patterned Sb₂S₃ film, a thin layer of CMC-N₃ is coated and locally exposed to UV light using a photomask to encode the information that exhibits controllable humidity responsivity. This hierarchical architecture enables the spatial separation of distinct information; each embedded at different vertical positions. At lower humidity levels, the information encoded on the Sb₂S₃ film is visible, while as the humidity level increases, the information encoded on the CMC-N₃ starts to reveal. This can be explained based on the humidity-dependent extinction ratio (ER) extracted from optical images using a contrast-based metric. To validate this concept, we used a transformative 7-segment display device to characterize the visibility contrast (Supplementary Fig. S9). The digit “2” is encoded on the Sb₂S₃ film; meanwhile, the digit “*5”* is embedded in the CMC-N₃ layer. The ER was defined as the ratio of local image contrast between humid and dry states, where contrast was calculated as the mean intensity difference between the digit region and its surrounding background (detailed information in Supplementary Note S1). Information encoded on Sb₂S₃ exhibits a decreasing ER with increasing humidity, indicating progressive loss of optical contrast at high RH. Meanwhile, the information written on CMC-N₃ displays a sharp increase in ER above 60% RH, attributed to humidity-induced swelling and enhanced optical modulation.

This image transformation in display design is further extended to images and QR codes encryption to validate the information storage capacity. Figure 4b demonstrates the multiplexing of two different image information, a UCSD Triton logo and QR code 1 (ndaolab.ucsd.edu) are imprinted on the Sb₂S₃ film using laser scanning, while the UCSD library logo and QR code 2 (ucsd.edu) are imprinted on the CMC-N_3_ layer using the UV exposure through a photomask. The information imprinted on the Sb₂S₃ film is readable with ambient room humidity (RH ~ 40%) while the information imprinted on the CMC-N_3_ layer remains hidden due to the similar thickness over the sample. As the humidity increases from 40% to 60%, the CMC-N_3_ layer responds, with regions having lower cross-linking density absorbing water molecules more quickly than the densely cross-linked hydrogel, thereby affecting the appearance of the information imprinted on the CMC-N_3_ layer. As the humidity reached 85%, the information encrypted in the CMC-N_3_ layer is fully readable (Supplementary video 1). With a further increase in humidity near saturation (RH ~ 95%), the hydrogel over-swelled, leading to shape distortion and rendering the information unreadable and deteriorated. Here, relatively larger pixel sizes are presented to showcase the versatility and capability of our method in fabricated functional devices, even with commercially affordable lithographic methods. Importantly, the lateral resolution in our system is not fundamentally limited by material processing. We achieved pixels as low as 3 μm (8467 PPI) as shown in Supplementary Fig. S10. Moreover, these materials are compatible with advanced nanofabrication techniques, such as UV nanoimprinting, enabling the formation of nanoscale structures and positioning them as promising candidates for next-generation nanophotonic platforms. The fabricated device was monitored over four months under ambient conditions without encapsulation. The Sb₂S₃ and CMC-N_3_ maintained the patterns, with only color degradation due to Ag nanoisland oxidation (Supplementary Fig. S11). We envision that the transformation of additional images can be enabled by exploiting the distinct refractive indices and swelling behaviors of individual layers within a multilayer hydrogel architecture. This could be achieved through advanced chemical strategies—such as incorporating different polymer types, tuning polymer crosslinking densities to modulate swelling responses, introducing non-swelling dielectric layers, or adding functional dyes to the polymer—to expand further the chemical programmability and design flexibility of the system. The system demonstrates high-resolution, full-color, reversible optical modulation using a single white-light illumination source, with independent layer addressability achieved solely through environmental humidity. This makes the platform suitable for multi-dimensional data encryption, rewritable anti-counterfeiting labels, and dynamic smart displays.

## Discussion

In conclusion, we have developed a novel stimuli-responsive optical data storage platform by synergistically integrating a phase-change Sb₂S₃ layer with a humidity-responsive CMC-N₃ hydrogel. This hybrid design enables reversible, full-color information encoding across multiple stacked layers—a capability previously elusive under incoherent illumination. Each material is independently programmable: Sb₂S₃ is written and erased via localized laser-induced phase transitions, while CMC-N₃ is patterned by UV exposure, allowing distinct data channels to be created and reconfigured within the same volume.

UV-patterned hydrogel functions as a static, permanent patterned layer, while the reversibility demonstrated in this work refers to humidity-triggered, reversible data retrieval/encryption (i.e., repeated conceal/reveal cycles enabled by reversible hydrogel swelling/deswelling). Rewriting is not performed by re-exposing the same hydrogel pattern; instead, device reconfiguration can be achieved by removing (washing off) the hydrogel and re-depositing/patterning a new hydrogel layer on the same substrate, while the Sb₂S₃ layer provides the rewritable storage medium through laser write and thermal/laser erase processes.

Critically, the multiplexed images remain readable under incoherent ambient light, and their visibility can be dynamically toggled by adjusting environmental humidity. This on-demand humidity-driven switching provides a unique mechanism for concealing or revealing data under ordinary conditions. The resulting multi-layer medium achieves high spatial resolution and sub-second response times, is fully rewritable, and maintains robust performance over repeated write–erase cycles and humidity modulation. While the UV-defined hydrogel patterns in our system are permanent and non-erasable due to irreversible crosslinking, the humidity-driven optical modulation they enable is fully reversible and repeatable. To expand the scope of hydrogel-based rewritable platforms, future developments could incorporate reprogrammable hydrogel chemistries based on dynamic supramolecular networks—utilizing reversible non-covalent interactions such as hydrogen bonding, host–guest inclusion, or metal–ligand coordination—or photo-cleavable crosslinkers that enable erasure and re-patterning through light exposure. Altogether, this multi-modal approach to optical memory opens new avenues in secure data encryption, anti-counterfeiting technologies, environmental sensing, and reconfigurable display applications.

## Materials and methods

### Synthesis of hydrogel

Photoreactive carboxymethyl cellulose-azido (CMC-N₃) derivatives were synthesized via carbodiimide-mediated grafting of phenyl azido groups onto the CMC backbone. Briefly, 600 mg of CMC (Mw = 250,000 g mol⁻¹) was dissolved in 60 mL of deionized water, and the pH was adjusted to 7.0 using 0.1 M NaOH or HCl. For CMC-N₃, the solution was reacted overnight at 4 °C with 108 mg of EDC, 108 mg of NHS, and 23 mg of 4-azidoaniline hydrochloride. All reaction mixtures were dialyzed against DI water for 72 h and then lyophilized to obtain the final CMC-N₃ products.

### Deposition of Sb₂S₃ thin film

The detailed flowchart of the fabrication process of our device is illustrated in Supplementary Fig. S12. The deposition of the phase-change material (PCM) platform based on Sb₂S₃ begins with the thorough cleaning of a BK7 glass substrate via sequential ultrasonication in acetone, isopropyl alcohol (IPA), and deionized water to eliminate organic residues and particulates. A 20 nm thin film of amorphous Sb₂S₃ (a-Sb₂S₃) is then deposited onto the cleaned substrate using RF sputtering at 100 W power in an argon atmosphere with a flow rate of 10 sccm, under a base pressure of ~1 × 10⁻⁶ torr. To protect the Sb₂S₃ layer from oxidation and sulfur loss, a 7 nm SiO₂ capping layer is subsequently deposited using RF sputtering at 200 W under the same vacuum and gas flow conditions. The stack is then annealed on a hot plate at 300 °C for 3 min to induce crystallization of the Sb₂S₃ layer (c-Sb₂S₃). Finally, spatially controlled laser irradiation is used to locally amorphized regions of the crystalline film, enabling reversible, high-resolution patterning for dynamic optical encoding applications.

### Data writing on Sb_2_S_3_ thin film

Switching Setup: A nanosecond pulsed laser (Quantronix Darwin Series) operating at a wavelength of 527 nm, with a pulse width of 150 ns and a repetition rate of 10 kHz, was used to induce amorphization in Sb₂S₃ thin film. The detailed optical schematic is shown in Supplementary Fig. S2. The beam passed through a polarizer (P) and a half-wave plate (λ/2) for power tuning, was then directed by dielectric mirrors (M) and combined with the white-light illumination path using a dichroic mirror (DM). The resulting beam was focused onto the sample using a 5× objective lens with a numerical aperture (NA) of 0.14, enabling high-resolution patterning with an effective laser power between 1–2 mW at the sample plane. For laser-induced crystallization (erasure), a 405 nm diode laser operated in continuous wave (CW) mode was used, with modulation controlled by a function generator and a maximum output power of 60 mW. Both lasers were aligned along a common optical axis and delivered to the sample through the same objective. The sample was mounted on a motorized 3D stage with 1 μm step resolution, allowing for precise spatial positioning during the writing and erasing processes. For imaging and real-time alignment, a 20× objective lens (NA = 0.42) was used in combination with an optical filter placed before the camera to block laser light. A camera positioned at the end of the system provided real-time imaging for alignment and feedback.

### Data writing on hydrogel

Before hydrogel coating and writing, Sb₂S₃-coated substrates were sequentially cleaned with deionized water, ethanol, and isopropanol, then dried under a nitrogen stream. For the adhesive layer, low molecular weight chitosan (CHI) was dissolved in 1.0 wt% aqueous solution containing 1.0% (v/v) acetic acid, followed by overnight stirring at 1000 rpm and 65 °C. The resulting CHI solution was spin-coated onto plasma-treated silicon wafers at 6000 rpm, then thermally crosslinked on a hotplate at 65 °C. CMC-N₃ layer was spin-coated at 2000 rpm. Photopatterning was achieved by UV exposure (15 mW cm⁻², 10 min) through a photomask, inducing crosslinking only in the exposed regions. Unexposed areas were subsequently removed by rinsing with water, leaving behind patterned crosslinked domains. After the UV patterning is completed, 25 nm of silver layer is deposited with electron beam evaporation under a base pressure of 3 × 10^−6^ Torr and a deposition rate of 1 Å/s.

### Characterization

The refractive indices of amorphous and crystalline states of Sb₂S₃ were measured with the J.A. Woollam M-2000D Ellipsometer. The optical transmission of the devices under varying RH conditions was measured using a specialized humidity chamber designed to control RH levels precisely, and the measurements were conducted at room temperature (20° ± 2 °C) using an Ocean Optics spectrometer with a white light source as the incident light. Response time, recovery time, and repeatability measurements were performed using a collimated white light source, photodiode (818-ST2-UV, Newport), and a power meter (1936-C, Newport) with a customized MATLAB data acquisition interface. The optical microscopic images were taken using a Keyence digital microscope (Keyence, VHX-1000).

### Simulation

The transmission spectra were simulated using the Finite-Difference Time-Domain (FDTD) method implemented in the commercially available Lumerical FDTD software. Ag nanoislands were randomly distributed with a Gaussian radius profile over a 1 μm × 1 μm simulation area. To ensure statistical reliability, the simulations were repeated 10 times with different random seeds. The coordinates of the silver (Ag) nanoislands were extracted from the atomic force microscopy (AFM) image by image processing technique using MATLAB. Effective refractive index values of the nanoparticle layer were extracted via S-parameter analysis using built-in functions in Lumerical and averaged over 100 iterations to account for structural randomness. The plane wave was incident on the structure with a wavelength range from 400 to 800 nm. Periodic boundary condition was employed in the x- and y-directions, and a perfectly matched layer (PML) was used in the z-direction. A uniform 3 nm mesh was utilized in the Ag particles-hydrogel-Sb_2_S_3_ structure. The refractive index input for the hydrogel layer incorporated its experimentally measured humidity-dependent variation, specifically calibrated to RH 40% conditions.

## Supplementary information

Supplementary information for Reversible Optical Data Storage and Encryption Enabled by Phase-Change and Hydrogel Integration

Illustration of scanning QR code

## Data Availability

The data that support the findings of this study are available from the corresponding author upon reasonable request.
