# Supplementary material for: Reversible optical data storage and encryption enabled by phase-change and hydrogel integration
Source: Light Sci Appl. 2026 May 18;15:238. doi: 10.1038/s41377-026-02330-5 (PMC13184341; doi:10.1038/s41377-026-02330-5)
Supplement: Supplementary file 1 — Supplementary information for Reversible Optical Data Storage and Encryption Enabled by Phase-Change and Hydrogel Integration [file 41377_2026_2330_MOESM1_ESM.docx]

Supplementary information

Reversible Optical Data Storage and Encryption Enabled by Phase-Change and Hydrogel Integration

Asad Nauman^†^, Guli Gulinihali^†^, Tristen Moncada, Muhammad Waleed Khalid, Tristan Tjussardi, Yeshaiahu Fainman, and Abdoulaye Ndao*

*^1^Department of Electrical and Computer Engineering*, *University of California*, *San Diego*, *La Jolla*, *CA 92093*, *USA*

^†^These authors contributed equally to this work.


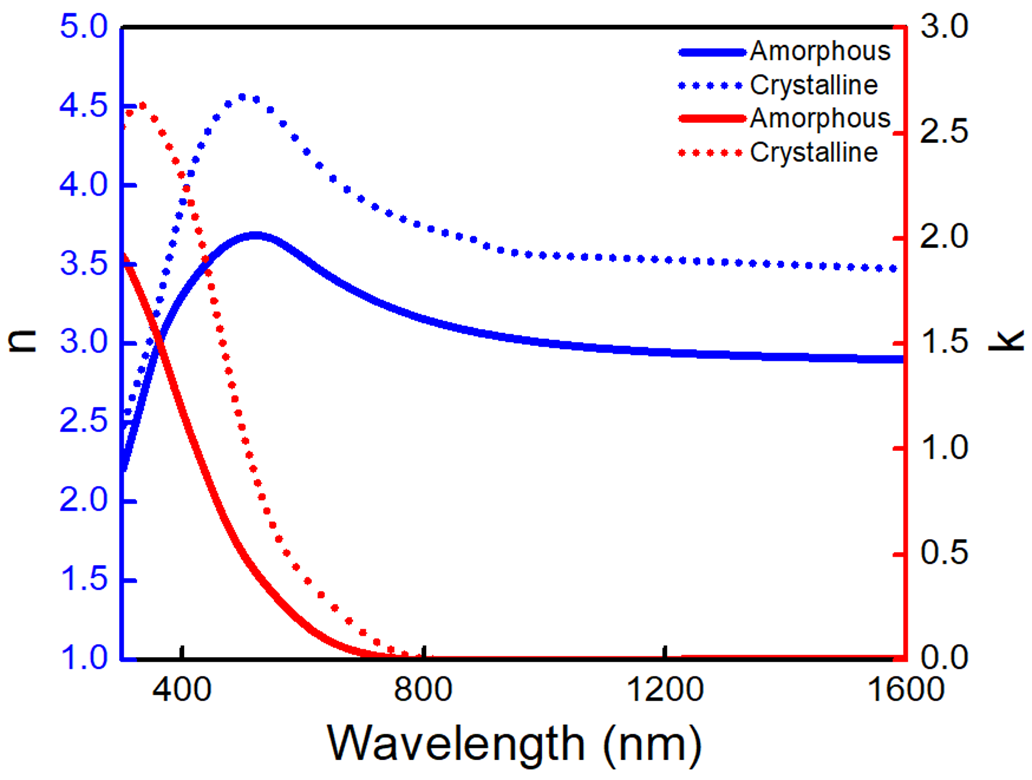


**Figure. S1:** Measured refractive indices “n” and extinction coefficient “k” of 20 nm thick Sb_2_S_3_ sample in amorphous and crystalline states.


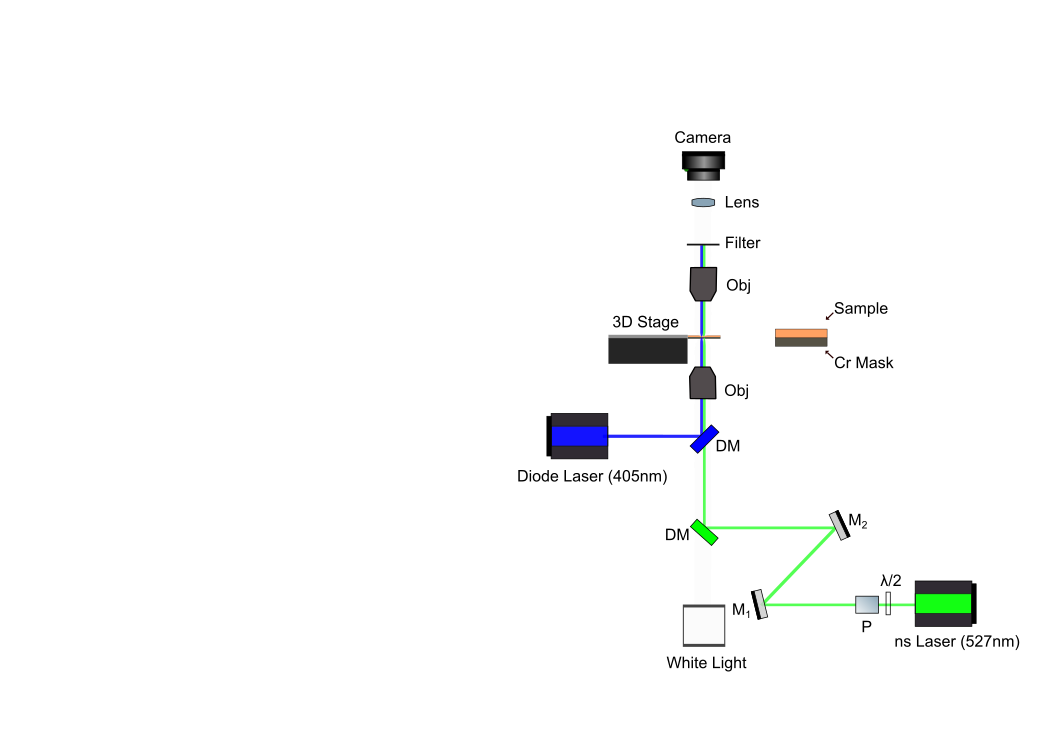


**Figure. S2:** Schematic of laser writing and erasing setup. λ/2: Half-wave plate. P: Polarizer. M: Mirror. DM: Dichroic mirror. Obj: Objective lens.


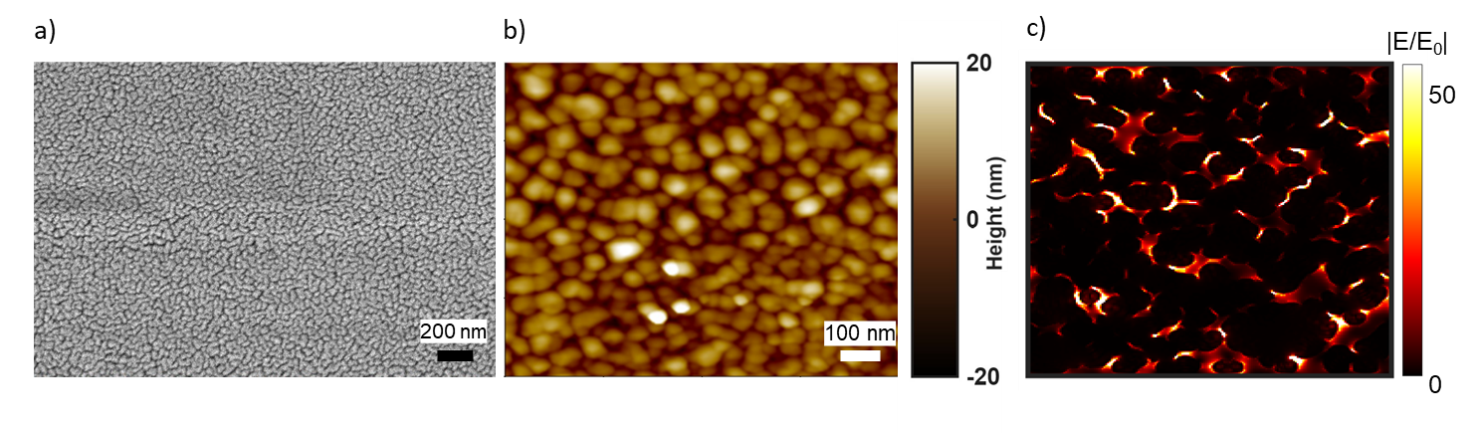
**Figure. S3:** a) SEM image and b) AFM image. c) Top view of simulated electric field magnitude profiles of Ag islands.


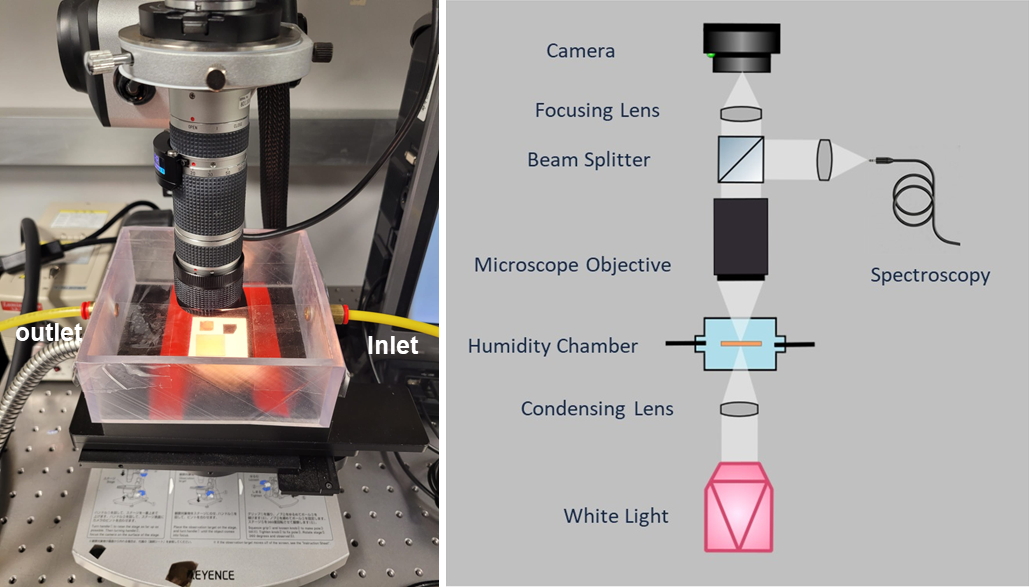


**Figure. S4:** Picture of the humidity chamber and the schematic of the hydrogel characterization setup


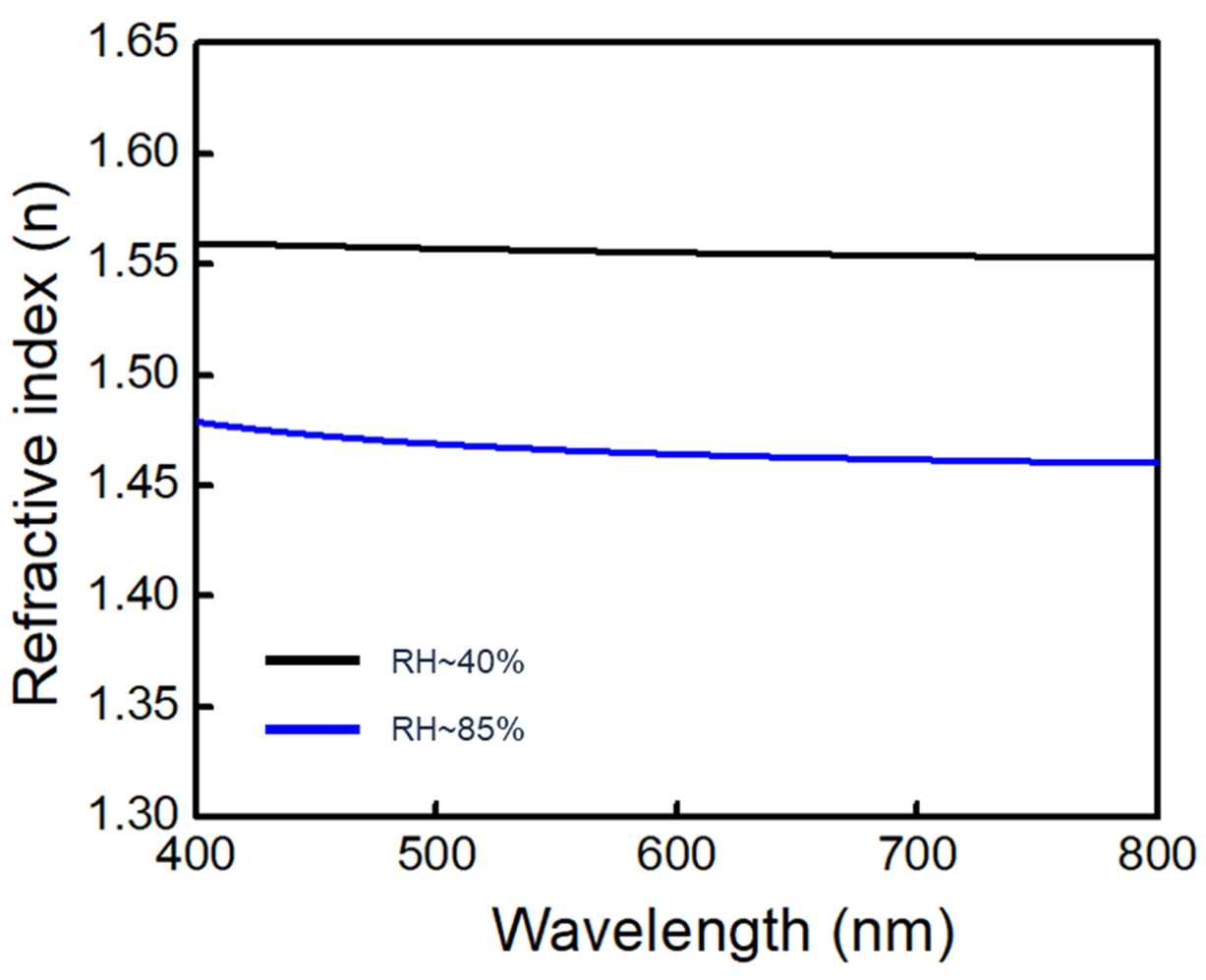


**Figure. S5:** Refractive index of hydrogel with different humidity. Black line: refractive index measured in 40% humidity. Blue line: refractive index measured at 85% humidity.


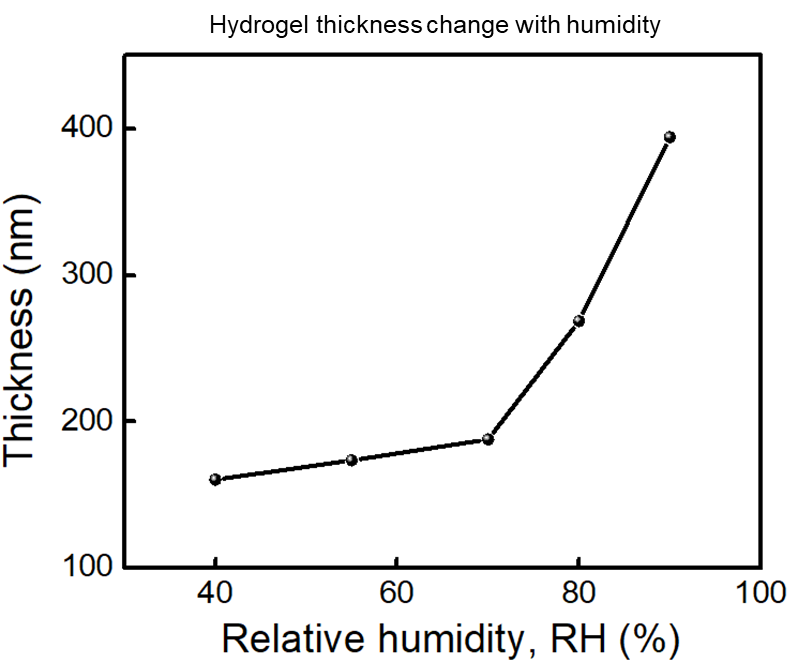


**Figure. S6:** Hydrogel thickness changes with different relative humidity.


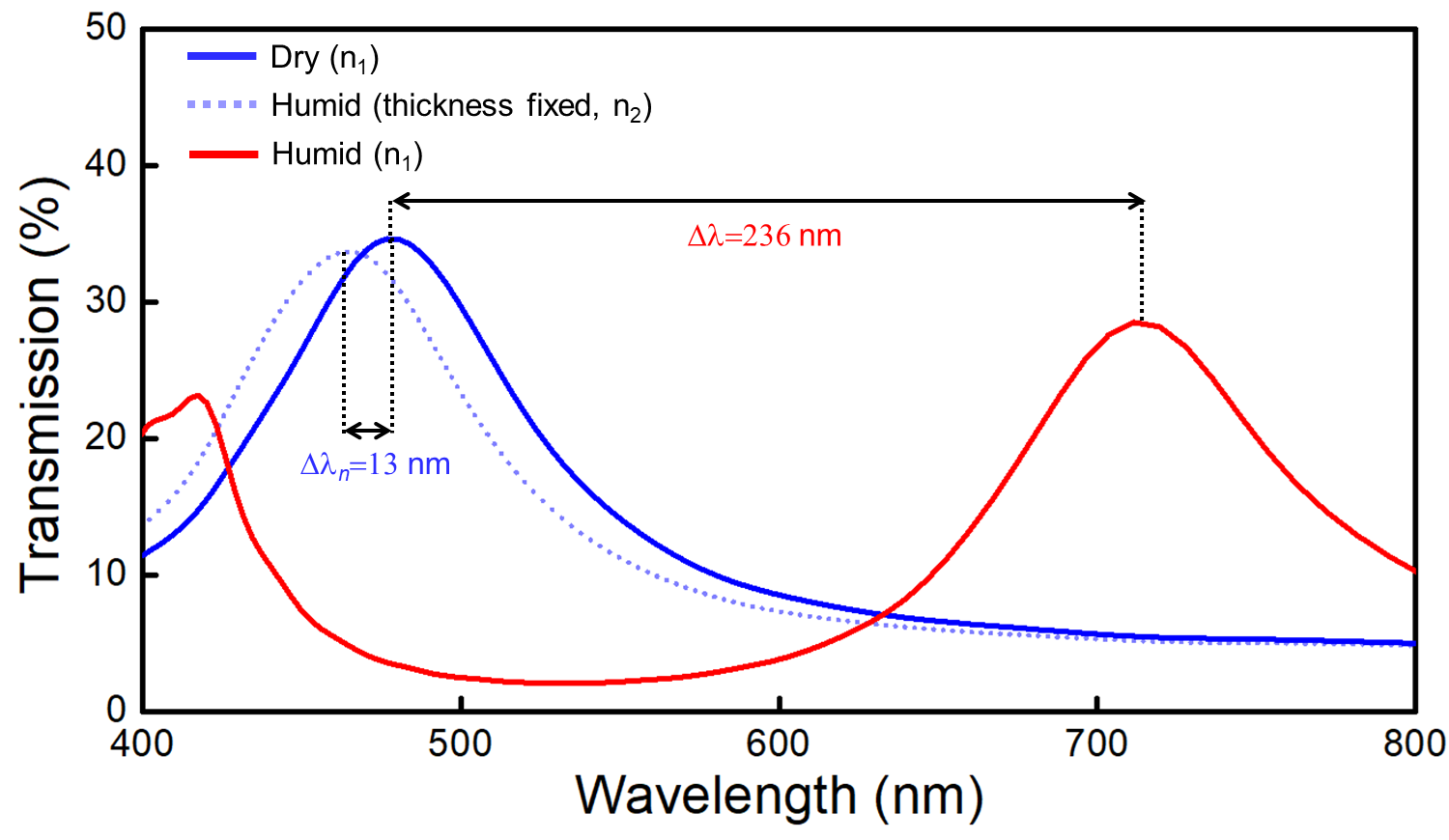


**Figure. S7:** Effects of thickness and refractive index change by increasing the humidity on the transmission spectra. Blue solid line: Dry (Initial state), Blue dotted line: Humid (only refractive changes by keeping thickness fixed), Red solid line: Humid, but the thickness is changed only, refractive indices remain same as initial state. Note, the refractive indices and thickness change considered in the simulations are taken from the data presented in Figure S5 and S6.


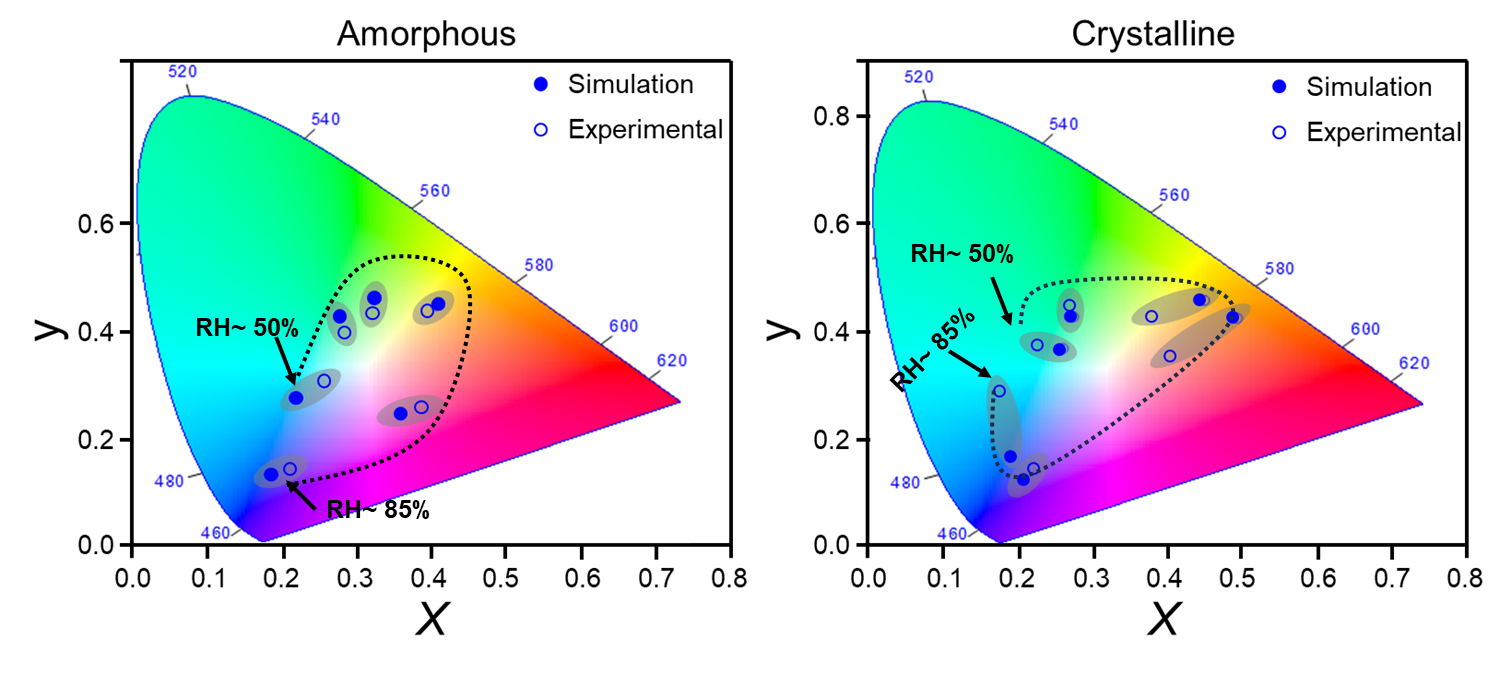


**Figure. S8:** Comparison of CIE chromaticity diagrams of the experimental and simulation results of the proposed device in amorphous and crystalline states.

Note S1:

The extinction ratio (ER) was calculated from images using a contrast-based definition acquired at different humidity levels. For each humidity state, the mean grayscale intensity of the digit region was compared with that of a local background surrounding the digit, yielding a contrast intensity value C = I_digit_−I_background_. The ER was then defined as the ratio of contrast between humid and dry states, ER = ∣C_humid_∣/∣C_dry_∣ and reported in decibels ER_dB_ ​= 10log_10_​(ER​). The decreasing ER of Sb_2_S_3_ with humidity indicates visibility decrease at high RH, whereas the increasing ER of the hydrogel reflects humidity-activated optical amplification, resulting in a crossover behavior near 70% RH. We employed a contrast-based extinction ratio, defined using local background subtraction, because it eliminates illumination non-uniformity, camera gain variations, and surface artifacts while directly quantifying local visual contrast in a manner consistent with human perception.


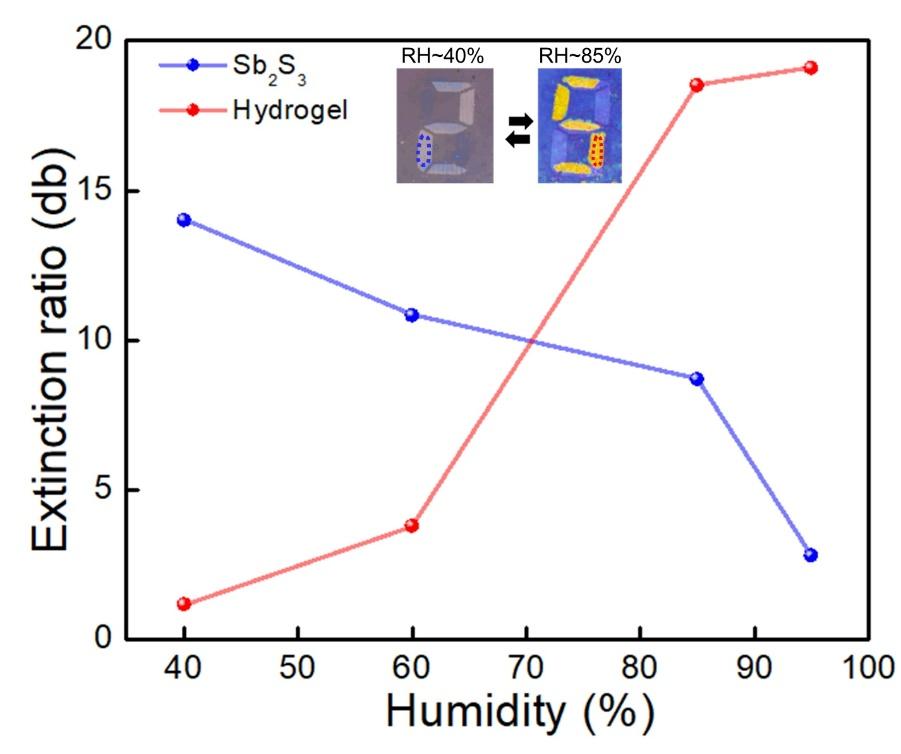


**Figure S9.** Extinction ratio as a function of relative humidity (%) for PCM-based (blue) and hydrogel-based (red) patterns. Blue and red dotted lines represent the area monitored to calculate the extinction ratio.


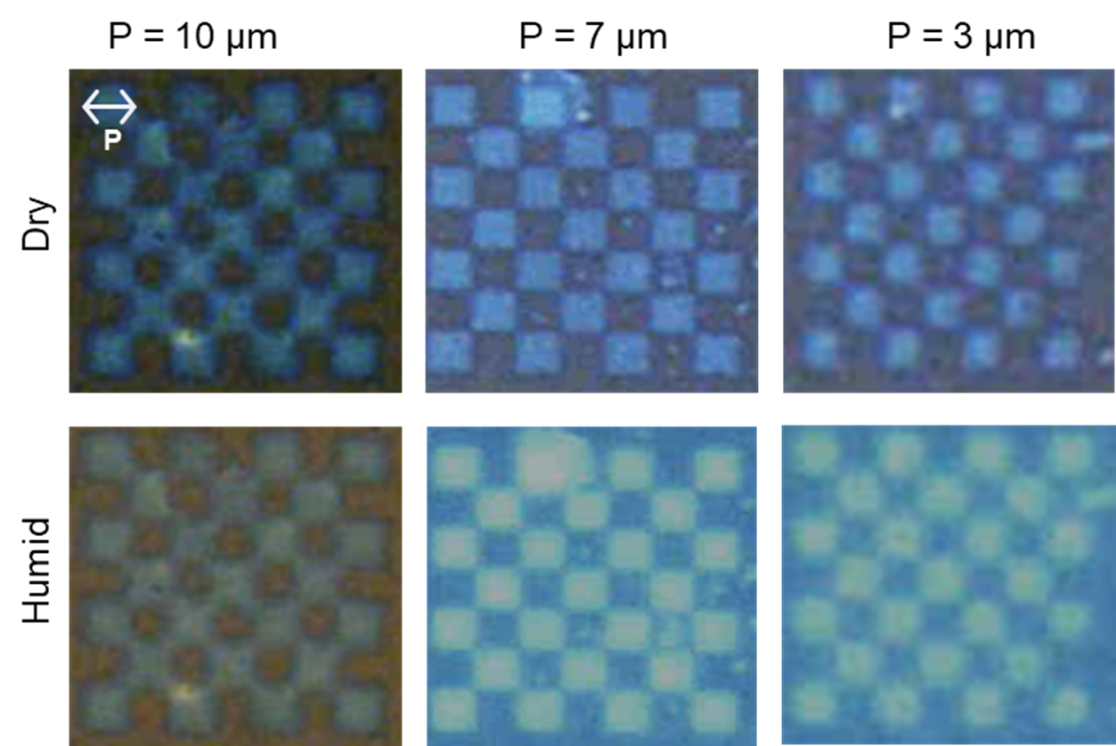


**Figure S10.** Resolution test pattern printed using MLA, featuring a checkerboard pattern in dry and humid state with pixel pitches of 10, 7, and 3 µm.


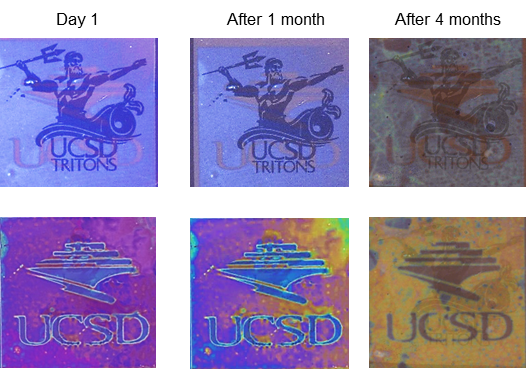


**Figure. S11:** Optical images of patterned regions acquired on Day 1, after 1 month, and after 4 months of storage under ambient conditions.


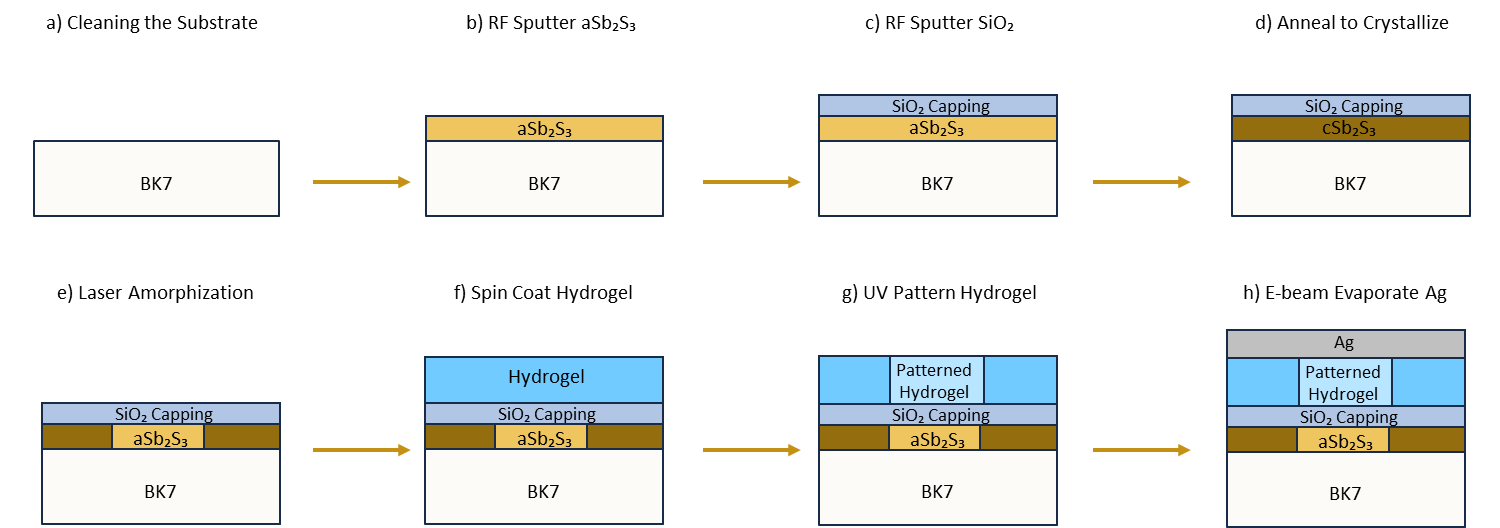


**Figure. S12:** The fabrication flowchart of the device platform.
